# Beyond the Genome 2000: The 18th International Congress of Biochemistry and Molecular Biology

**DOI:** 10.1002/1097-0061(200012)17:4<314::AID-YEA47>3.0.CO;2-K

**Published:** 2000

**Authors:** Jo Wixon, Francesco Brancia

**Affiliations:** 1 School of Biological Sciences University of Manchester Stopford Building Rm. 2.205. Oxford Road Manchester M13 9PT UK; 2 Michael Barber Centre for Mass Spectrometry UMIST, PO Box 88 Manchester M60 1QD UK

## Abstract

The meeting was held on 16–20 July 2000 at the International Convention Centre in Birmingham, UK, and was co-organized by the International Union of Biochemistry and Molecular Biology (IUBMB) and the Federation of European Biochemical Societies (FEBS). Although the meeting had a broad subject area, the emphasis was firmly placed on post-genomic studies, and hence several sessions should be of interest to our readers. We provide highlights of these sessions, bringing you a report on the most exciting and informative presentations.


**Yeast**
*Yeast* 2000; **17**: 314–321.

### Meeting Highlights

### Beyond the Genome 2000: The 18th International Congress of Biochemistry and Molecular Biology http://www.iubmb2000.org/

Jo Wixon[1]* and Francesco Brancia[1,2]
[1] *School of Biological Sciences, University of Manchester, Stopford Building Rm. 2.205. Oxford Road. Manchester M13 9PT, UK*
[2] *Michael Barber Centre for Mass Spectrometry, UMIST, PO Box 88. Manchester M60 1QD, UK*

*Correspondence to: Jo Wixon, School of Biological Sciences, University of Manchester, Stopford Building Rm. 2.205, Oxford Road, Manchester M13 9PT, UK*

Received: 19 September 2000
Accepted: 19 September 2000

### Abstract

**The meeting was held on 16–20 July 2000 at the International Convention Centre in Birmingham, UK, and was co-organized by the International Union of Biochemistry and Molecular Biology (IUBMB) and the Federation of European Biochemical Societies (FEBS). Although the meeting had a broad subject area, the emphasis was firmly placed on post-genomic studies, and hence several sessions should be of interest to our readers. We provide highlights of these sessions, bringing you a report on the most exciting and informative presentations. Copyright © 2000 John Wiley & Sons, Ltd.**

### Plenary talks

The first presentation of the meeting (the Severo Ochoa Lecture), entitled **'Decoding of the Human Genome'**, was given by Craig Venter (Celera, USA). After giving an overview of the progress made with sequencing using fluorescent dyes, he went on to discuss Celera's efforts to sequence the 'human genome'. Samples were obtained from three females and two males, two of whom are Caucasian, one is Hispanic, one is Chinese and one is African-American. The sequence to date (produced from one of the males) has an average of 45-fold coverage and will be submitted for publication by the end of the year, simultaneously with the results of the public project. The genomes of the other individuals are still in progress. High quality stretches of the sequence are already being used to define SNPs with ca. 6 million currently in their database.

Celera had already sequenced 2.9 billion base pairs of the mouse genome by the time the meeting started and they aim to have it completed by December. They see using the mouse genome as an aid in human gene prediction as easier and much more efficient than trying to get large numbers of cDNAs sequenced.

A new direction for Celera is proteomics, with the initiation of a Human Protein Anatomy Project. This will involve high-throughput proteomics studies using vastly improved mass spectrometers and large-scale production of antibodies against human proteins.

The meeting ended with George Poste of SmithKline Beecham (UK) giving his view of molecular medicine, population genetics and the **future of healthcare delivery** in the Datta Lecture. All of the drugs produced so far in the last five years act on just 450 targets, and the seven major classes of antibiotics are directed towards only 13 targets. Much of this could be due to the reliance on *in vitro* techniques, so it is his hope that the application of such approaches as microarrays will allow researchers to access other targets. The arrays allow observation of gene expression inside the host and give the ability to compare infection of different tissue types. They can also be used for diagnostic purposes, for example, they have been used in attempts to define molecular genotypes of various cancers. He also sees huge potential for pharmacogenomics, not only in the tailoring of medicines, but also in the avoidance of side-effects. They plan to construct chips representing all the class I and II metabolic enzymes, to allow patient profiling for response to drugs, and he thinks that this could realistically be available in less than 5 years. Disease stratification is a tougher nut to

Copyright © 2000 John Wiley & Sons, Ltd.

crack, which he thinks will take 5–10 years. Unconvinced as yet by current gene therapy approaches, he is particularly concerned about moves towards the production of 'stealth vectors' that would evade our immune system. He is more excited by the ideas that have emerged since the production of Dolly (the sheep), namely reprogramming gene expression, with a view to regenerative medicine, especially in neurons. He hopes that due to all these approaches, we will see a move towards more preventative medicine, rather than the reactive medicine we have now. His final comments took in the handling of data, with a call for more widespread adoption of nomenclature systems and more careful construction of databases, including more thorough annotation.

### Genomes and their analysis

Byrappa Venkatesh (Singapore) discussed the potential of the pufferfish ***Fugu rubripes* as a model organism**. The *Fugu* genome is about seven times smaller than the human genome and it encodes a similar gene repertoire but contains much less 'junk', making genes and regulatory regions easier to find. He presented work using *Fugu* to aid the detection of human gene regulatory regions. By using sequence comparison it is possible to predict regulatory regions. By introducing a *Fugu* cosmid, in his particular example encoding oxytocin, into a mammalian cell line, it was possible to demonstrate that the predicted regulatory sequences were functional and adequately conserved to allow expression of the *Fugu* gene.

Thomas Hudson (Montreal, Canada) gave a presentation on the state of **human genetics** studies, commenting on the effects of the draft human sequence and the SNP consortium data. There are currently 120 000 polymorphisms in the databases, and they hope this will be increased to 300 000 in the next 6 months. These polymorphisms can aid genetic screens and then become candidates for the disease-causing polymorphism.

Another powerful tool for genetics is the ability to use founder populations, which are derived in from a small pool of founders. These kindreds therefore have a limited number of alleles for each gene and can more easily be used to trace the inheritance of genetic diseases. There are several classic founder populations, for example, there is a very old Finnish kindred (2000 years), there are the Saguenays of Quebec, and the Ashkenazim. ARSACS disease, a progressive ataxia, is an example of a disease where the gene, Sacsin, was identified by positional cloning after genetic analysis of a kindred.

In contrast, the draft sequence is not always so widely useful. In the case of his group's asthma study, their genetically defined region of interest fell into an area neglected by the sequencers, so that although there was some sequence for the region, it was not contiguous. As a result, to characterize the region, his group had to generate clones, sequence them and perform their own annotation of the region.

### Comparative and evolutionary genomics

Peer Bork (EMBL, Germany) presented several approaches to **comparative genomics** being explored at EMBL. The first uses the number of orthologues between two species to estimate the evolutionary distance between them (allowing for the fact that the probable number of orthologues rises with genome size). The results of these analyses have been used to draw new evolutionary trees, which are less affected by lateral gene transfer. A second approach, called 'differential genome display', looks at which genes present in genome one (say, *Haemophilus influenzae*) are not present in genome 2 (say, *Escherichia coli*). This has been used to identify virulence genes shared by *H. influenzae* and *Helicobacter pylori* and absent from *E. coli* and has shown that these two pathogens share different complements of metabolic genes with *E. coli,* which could be due to differential evolution, allowing them to cope with their specific environments. An extension of these comparisons is the production of profiles of presence or absence for any gene, across a range of species. These results, coupled with searches for conserved neighbouring genes, shared regulatory regions and observation of gene fusions are being used to predict the function of ORFans.

Ken Wolfe (Dublin, Ireland) posed the question, 'Is **eukaryote gene order** truly random?'. The high frequency of inversions during gene order evolution in eukaryotes has certainly made it seem so, and as yet very little data exists in this area. Using *Candida albicans* data from a whole genome shotgun done at

Copyright © 2000 John Wiley & Sons, Ltd. 

Stanford and from cosmids sequenced at the Sanger Centre gave ca. 90% coverage of the genome, allowing detection of 275 conserved neighbours, representing only 10% of the *Saccharomyces cerevisiae* neighbours. Of these, 109 are inverted, from this he estimates that ca. 1200 single-gene inversions have occurred since speciation. These single-gene inversions should lead to genes 'waltzing' along chromosomes. A further 62 *S. cerevisiae* neighbours were separated by only one or two genes in *C. albicans,* in fact the bulk of neighbours are separated by small numbers of intervening genes, supporting the 'waltzing' hypothesis, over large-scale rearrangements. He has obtained similar results looking at *Arabidopsis thaliana*, finding evidence for many small inversions by comparing gene order in the large duplicated regions. In *Caenorhabditis elegans* a similar result was obtained by making use of the high level of intra-chromosomal duplications. Tandem repeats originate as two neighbouring copies of a gene, with the same orientation. He has shown that the further apart the two copies are, the more likely they are to be inverted, which indicates that 'waltzing' has taken place. A similar result was obtained by examining long-range *Fugu* sequence from 21 cosmids (McLysaght *et al.*, [2000]).

### Functional genomics

Guy Oshiro (Stanford, USA) presented an overview of **parallel functional analyses of the *S. cerevisiae* genome**. The first approach utilized Affymetrix chips, to assess global gene expression levels in yeast cells grown under nine different conditions. The MMS and UV response induction conditions gave the most similar results, showing common induction of proteasome genes, proteolysis genes and cytoplasmic degradation genes. A search for over-represented sequences in the upstream regions of these genes identified an Rpn4 binding site in 28 of the 178 genes. A second study involves expression profiling of 900 potential nORFs (non-annotated ORFs, predicted to encode proteins of $<100$ amino acids). Several nORFs showed identical expression patterns to larger upstream ORFs, indicating that these may be another exon of these genes or that they share, or have similar, regulatory regions. However, a significant proportion showed independent expression and they plan to further study these genes. In a further attempt to assign function to yeast genes, a large consortium of laboratories has produced oligo-tagged deletions of every yeast gene. In this way, a chip loaded with the tag oligos can be used to monitor the presence or absence of any mutant in a population before and after a phenotypic test. These chips have been used with samples from yeast populations grown under a range of conditions and the genes are then clustered according to their phenotype profiles. The tagged strains and tag sequences are now publicly available.

Marc Zabeau (Gent, Belgium) presented a study on **genome-wide expression analysis in plants**. His group have applied AFLP techniques to cDNA-based transcript profiling. The cDNA is digested with particular enzymes to produce unique 3′ end tags (they have used MseI, with BstYI or ApoI), and linkers are ligated to the ends of the fragments. The products are then amplified with primers complimentary to the linkers, with one or two extra bases, which then specify a fraction of the total products, facilitating amplification of less common fragments. This technique has enabled them to observe poorly expressed genes and to distinguish members of gene families. However, it is laborious, requiring many PCRs per sample and sequencing of re-amplified AFLP tags to allow identification. A test project investigated expression of genes in tobacco cell suspension culture at 12 time points during the cell cycle. Over 1000 tags were shown to be modulated during the cell cycle, most of which are expressed during the $M–G_1$ transition. From the 800 sequenced so far, more than half have no significant database match. Those that were known cell cycle genes were shown to have the expected expression profiles, validating the technique. A second approach is microarray analysis of the expression of *Arabidopsis* genes. The array has been loaded in duplicate with 4608 cDNA clones from the Incyte UNIGEM library, which should cover around one-fifth of the *Arabidopsis* gene complement. However, over 60% of *Arabidopsis* genes are parts of families, so the group, along with German and French collaborators, are currently making a large number of gene-specific probes for loading onto the final 26 000 gene array.

László Patthy (Sopron, Hungary) gave a presentation entitled **'Unique Functional Composition of Metazoan Genomes'**. The observation that, in prokaryote genomes, gene density is limitingly high

Copyright © 2000 John Wiley & Sons, Ltd. 

and genomes with gene densities of over 500 genes/megabase have no introns in their genes lead him to the question, 'What effect do introns have on evolution?'. They allow differential splicing and, he would argue, introduce intronic recombination as an evolutionary mechanism. Introns of the same phase can shuffle without changing the frame of the gene, and new exons can be introduced, so long as their introns have the same phase. He has found evidence for this mechanism in many animal genes, such as plasminogen and fibronectin, and can also see some correlation between domains of structure and exon boundaries (this is aided by observing multiple species, since some introns have been lost in some lineages). One important distinction is that intracellular protein domains (also found in bacteria) do not shown much evidence of exon shuffling, whereas extracellular protein domains, particularly those that show clear evidence of exon shuffling, are found only in animals, indicating that this mechanism could be specific to animals. His idea is that when spliceosomal introns invaded animal genes, they started making domains and shuffling. The genes showing evidence of shuffling encode modular receptors, cell adhesion- or membrane-associated proteins, all of which are specifically required by multicellular organisms for purposes of communication and adhesion. More detailed observation shows a proliferation of this mechanism in the vertebrate lineage. This mechanism appears to have started at the time of the metazoan radiation and to have been maintained throughout chordate evolution. There are some intracellular genes that show exon shuffling, but he argues that these are 'young' genes, since they have a good correlation between domains and exon boundaries, which is typically lost over long periods of evolution.

### The proteome and beyond

David Eisenberg spoke about approaches his group have used to **assign function to proteins predicted from genome sequence**. The first method has been called the 'Rosetta stone' method, where observing a gene appearing as a fusion in one species can be used to predict the function of the gene in another species. Using this method, they have found 45 000 links in other organisms for genes from yeast. It is very common to see fusion of genes with functions in the same metabolic pathway, for example, the entire purine biosynthesis pathway gene complement have been found as fusions in one or more species. A second approach is phylogenetic profiling, where genes are categorized according to co-occurrence in a wide range of species. This method has so far been used to generate 20 749 links for yeast proteins. However, the technique is limited in that it is not informative for genes unique to one species or for those genes found in all organisms.

The group have found that a combination of the two methods gives the best results, those genes with a matching prediction from the two methods having a much higher chance of being correctly linked. Data from these and other studies linking proteins together can be found at **http://dip.doe-mbi.ucla.edu**

In his talk entitled **'Defining Gene Function via Mass Spectrometry Analysis of Multiprotein Complexes'**, Matthias Mann (Odense, Denmark) described the typical approach employed for large-scale mapping of multi-protein complexes. The complex is biochemically purified using an antibody or a tagged member of the complex. The proteins are subsequently separated using two-dimensional gel separation and the spots are excised, enzymatically digested and the resulting, unpurified mixture micropurified and analysed by nanoelectrospray tandem mass spectrometry.

The human spliceosome machinery was the first large mammalian complex purified in this way (Neubauer *et al.*, [1998]): many known proteins and novel-splicing factors were isolated and identified by mass spectrometry among the 70 proteins that constitute the complex. The identification of all novel proteins was performed using expressed sequence tag databases and most of them were then cloned and functionally studied. This method has been successfully applied to several different cases: for instance, various signalling complexes have been investigated. In the complex involved in the ECF receptor pathway the protein complex was isolated using phosphotyrosine as antibody tag.

### Applications and exploitation of systems approaches to gene function

Steve Oliver (Manchester, UK) described the progress made by his group in the **functional analysis of the yeast** ***Saccharomyces cerevisiae***. Initial transcrip-

Copyright © 2000 John Wiley & Sons, Ltd. 

tomics studies involved Northern blot analysis of 1000 ORFs under a range of conditions. They have studied the correlation between this data and the clusters produced from the DeRisi *et al.* ( [1997]) data. For many clusters, the comparison was quite good, but differences between the normalization systems used caused problems and it was not possible to compare data for some pathways. This lack of ability to compare data between methods is a significant concern in transcriptomics. Another observation from the expression data was that genes overexpressed in response to glucose upshift had a high CAI, whereas in stationary phase or nitrogen starvation there is no such correlation with CAI. This could be because under these conditions, cells have excess translational capacity.

Proteomics studies include an improvement upon the number of tryptic peptides which can be detected by mass spectrometry using a derivatization step, in which N-terminal lysine residues (which are less easily detected) are converted to homo-arginine (Brancia *et al.*, [2000]). Combining the data from this method with Edman degradation and searches of a database of all predicted *S. cerevisiae* tryptic peptides (PepMAPPER: **http://wolf.bi.umist.ac.uk/mapper**) greatly improves the chances of a correct identification.

A new study involves the screening of mammalian cDNA libraries to identify functional homologues of essential yeast genes. This will be done using downregulation of yeast essential genes by *tet*O, coupled with transformation with mammalian cDNAs under the control of a MET3 promoter. This allows controlled switching between expression of the native yeast gene and the mammalian gene.

Ronald Plasterk (Amsterdam, The Netherlands) spoke about **RNA interference and transposon silencing in *C. elegans***. *C. elegans* has three types of transposons, TC1 (mariner), TC3 and sleeping beauty (which exists only as remnants). It was observed that one strain (Bristol) showed no movement of these transposons in its germline. This could not be due to lack of a transposase, since TC1 and TC3 each have their own TC-specific enzymes. To find out what was causing this, they made a strain with a TC1 insertion into myosin, rendering it non-motile. Ethylnitrosourea (ENU) mutagenesis of this strain would produce motile worms if transposition were to be recovered. Analysis of several of the 43 motile mutants showed mutations in a homologue of RNAse D. Over half of the mutants (including the RNAse D mutants) were resistant to RNAi, prompting his theory that RNAi could be a natural antivirus mechanism. Once a virus has moved around enough, read-through of nearby genes can cause production of both strands of the virus, initiating RNAi against the viral mRNA. This blocks any further transposition, but leaves the integrated copies intact. This could be because it is too risky to target parts of the genome, although in plants RNAi also blocks transcription by methylating the matching genes.

Andre Goffeau (Louvain-la-Neuve, Belgium) presented the progress made by his group towards the **development of new fungicides using functional genomics approaches**. There are 20 predicted yeast ABC transporters of varying topology, Andre's group have concentrated on the double transmembrane span ones, of which there are two families, with reverse topology. It was known that mutations in a transcriptional activator, PDR1, caused multidrug resistance. They have shown that in a pdr1p mutant, the PDR5 and SNQ2 ABC transporters are overexpressed and that a third ABC transporter, YOR1, is only expressed when PDR1 is overexpressed. Comparing wild-type PDR5 and mutant pdr5 strains showed that this transporter confers resistance to a huge range of compounds, including antibiotics, detergents and ionophores, as do SNQ2 and YOR1. To find out which other genes are regulated by PDR1, a microarray was used to compare wild-type PDR1 against mutant pdr1 yeast. Twenty new genes that are activated (and several which are downregulated) by PDR1 were found in this way. The activated genes include permeases, new ABC transporters and genes involved in stress response and cell wall composition. A triplicated study of the proteome of pdr1pdr3 mutants revealed 41 spots that decreased in intensity compared to wild-type. These results did show some agreement with the microarray data, but also detected changes at the protein level that were not the result of changes at the level of mRNA. However, they feel that the two approaches are complementary, since they can only see changes in membrane proteins using microarray. Yeast has homologues of bacterial $H^+$ antiporters as large families. Deletions of some of these have also been made, and the mutants do have increased drug sensitivity.

Copyright © 2000 John Wiley & Sons, Ltd. 

### New technologies: genomics and proteomics

David Hunt (Charlottesville, USA) presented a different proteomic approach in his talk entitled **'Proteomics: Automated Identification of Peptides and Proteins at the Attomole Level in Complex Mixtures by Mass Spectrometry'**. The proteins present in the complex are isolated using chromatographic techniques and the resulting fractions are analysed using electrospray quadrupole ion trap mass spectrometry, providing protein identification up to attomole level. The fractionation of the mixture by two-dimensional electrophoresis is not required and characterization of post-translational modification is facilitated.

Many examples were presented from the analysis of (a) protein–protein interactions that regulate a human phosphatase; (b) proteins that enable a plant to synthesize its own fungicide; (c) antigens presented by class II MHC molecules; and (d) proteins secreted by tumours.

### Genomics in agriculture and medicine

Mike Bevan (Norwich, UK) reported on the **findings from the *Arabidopsis* genome**, which is now nearly completed. At the time of his talk, they had sequenced 95–97% of their BAC clones, which amounted to 125 Mb. Chromosomes 2, 4 and 3 were complete, with chromosomes 1 and 5 in the finishing stages. Cereon have donated 39 000 SNPs to be integrated into the map, from their work with the Landsberg erecta line. *Arabidopsis* genes are closely packed, which can cause the exon prediction programmes to concatenate genes when they build 'gene models'. MIPS, Kazusa and TIGR are all independently doing the annotation, the aim being to increase the confidence of their predictions when they all agree. They are also using cDNA sequences to aid in gene prediction and so far the gene models agree for 90% of the genes; 10% of the genes have experimentally defined function, 50% have significant homology to genes from another organism and 40% have no significant homology to any gene from another organism. The distribution of the genes across functional categories is quite different from animal genomes; the 'defence' portion is much larger, which is probably because plants have no option to move to evade a stress. A huge proportion of genes are involved in metabolism and transcriptional control. On average, plant metabolic genes are more likely to be similar to algal genes than to animal ones; however, there are several genes (e.g. from the transcriptional control category) that are highly homologous to their animal counterparts, despite the billions of years that have passed since these lineages diverged. A functional genomics project is planned in the UK; a network of labs (called GARNET; see: **http://www.york.ac.uk/res/garnet/garnet.htm**) will be involved in transcriptome, proteome and metabolome studies, using the BAC clones for complementation studies, and making ca. 30 000 transposon insertion lines.

Julio Celis (Aarhus, Denmark) gave a talk on the use of **proteomics strategies in the study of bladder cancer**. His first comment was that he feels that two-dimensional gel electrophoresis is still the way to go for proteomics, and that he has yet to be convinced by the other ideas currently being discussed. Whilst the gels are not such an issue, sample preparation is very important. Tissue samples are difficult to work with because they can contain too many cell types to give meaningful results and tissue culture cells are not exactly like fresh biopsy material, so protein levels can vary. Another hurdle is the level of expression of the proteins of interest. They have had to hybridize antibodies to blots of their gels to observe many of the known oncogene products and other proteins they were interested in, due to their low abundance. They have started to produce a database of their results, by scanning the gels and assigning numbers to each spot. Once this is done, they can annotate the gel, perform quantitation and compare to other gels (see: **http://biobase.dk/cgi-bin/celis**). Their first aim was to produce a result for normal bladder tissue, which was done by combining the results from four samples. Next they dissected out bladder tumours and compared the gels to identify genes expressed only in tumour cells and genes expressed only in normal cells, to act as markers for each cell state. They then dissected tissue in sections progressing towards a tumour, and showed that they could see a clear boundary of transition to tumour state, at some distance from tissue that clearly looked cancerous. In addition one of the tumour markers, psoriasin, is excreted, so now they can use this to make a urine test by running a 2-D gel of urine and hybridizing the blot with antibody.

Copyright © 2000 John Wiley & Sons, Ltd. 

Tim Harris (La Jolla, USA) gave an overview of **the application of high throughput X-ray crystallography to drug discovery**. Whilst there are more than 12 000 entries in PDB, only ca. 2500 unique structures have been determined. Another concern is the growing number of cases of proteins having very different sequences but showing the same fold structure, indicating that we can no longer lean on sequence-based structure predictions. A classic example of the contribution that a structure can make is that of Tubby (a mouse obesity protein family member). The structure contained a basic cleft, which was predicted to be a DNA-binding domain and was found to correlate with the position of known functional mutations. This then led to band shift experiments and subcellular localization studies that confirmed the DNA-binding factor prediction.

The current technology available includes third-generation synchrotron sources, MAD phasing and selenomethionine labelling of proteins, CCD detectors and cryoprotection of crystals. Approaches aimed at improving throughput take in automation of cloning and expression phases, high throughput affinity purification steps, microcrystallization in 96- or 384-well plates, and image analysis methodologies. At Structural Genomix they are running an antibacterial programme in which they take the complete gene complement of a bacterium, subtract the PDB hits, in particular those with human homologues, and then look at bacterially conserved genes as potential targets for broad-spectrum drugs and at organism-specific genes as potential targets for niche drugs. If the gene of interest is found in several species, their approach is to purify, and try to crystallize, all known orthologues and, if possible, to compare the structures obtained.

### New technologies: structure to function

Advanced atomic force microscopy (AFM) of biological samples was described by Andreas Engel (Basel, Switzerland) in the presentation entitled **'Progress in Biological Atomic Force Microscopy'**. The state of the art of this technique and manipulation of bacteriorhodopsin (BR) were reported. The light-driven proton pump in the cell membrane of *Halobacterium salinarium* is packed in a highly ordered 2-D trigonal lattice in the purple membrane. Immuno-AFM of purple membranes was employed to characterize the two distinct BR surface topographies with a lateral resolving power of 5 Å and a vertical resolution of 1%. This technique enables the determination of the conformations of the native BR surfaces and the mapping of the flexibility of individual polypeptide loops connecting the transmembrane helices. A comparison with structural data obtained from electron and X-ray crystallography demonstrated the accuracy of mapping by AFM.

The prospective application of LINAC-driven free electron lasers in biological research was reported in the talk **'Scientific Potential of X-ray Free-electron Lasers'**, presented by Jochen Schneider (Hamburg, Germany). Protein crystallography is a growing field of synchrotron radiation research that in 5 or 10 years will be changed by the introduction of these new types of storage rings. LINAC are new radiation sources that they expect will produce spatially, fully-coherent X-ray beams with an average brilliance of about 6 orders of magnitude higher than the third-generation synchrotron radiation facilities. Since the pulse duration (frequency) would be reduced from 50 ps to about 100 ps, this should provide a peak brilliance of about 10 orders of magnitude higher than what we use today. This X-ray laser represents a big opportunity for the physics community, and also offers a powerful tool to those investigating biological systems.

### Genetic analysis of embryonic development

Derek Stemple (London, UK) presented work on **mutations affecting embryogenesis in zebrafish and *Xenopus tropicalis***. The notochord forms the basis for the nervous system and in the 12–24 hour zebrafish embryo, undergoes a dramatic transition into differentiated cell types. The genes known to affect this transition all result in short, dwarf fish, with no vacuolated notochord, which fail to stop expressing some early marker genes. Positional cloning of two of these genes, *grumpy* and *sleepy,* is under way; *grumpy* is tightly linked to myoD and *sleepy* is on linkage group 2, in regions for which they have PAC and YAC clones, respectively. These mutants have all come from the Tübingen and Boston genetic screens of zebrafish. To assess whether saturation has been reached for this path-

Copyright © 2000 John Wiley & Sons, Ltd.

way in the zebrafish, he compared the two screens to work out the mean number of alleles per locus. Using a Poisson distribution he has then estimated the number of loci in the zero-allele class (i.e. not detected). From this, he estimates that notochord genes have been saturated in these screens. This prediction is also supported by complementation testing between the two screens. So, the next move, for a consortium of laboratories (see: **http://minerva.acc.virginia.edu/~develbio/trop/**), is to start looking in a different model organism, *Xenopus tropicalis*. This frog has a ca. $1.7 \times 10^9$ bp diploid genome, spread over 10 chromosomes, and a shorter generation time (only 3–4 months) than *Xenopus laevis*. It is easy to make transgenic lines and there is already an efficient insertional mutagenesis methodology. The NIH will be funding an EST project, the production of large insert genomic libraries, radiation hybrid panels and meiotic maps.

Julie Ahringer (Cambridge, UK) reported on work towards **'Linking the genomic sequence to embryonic patterning in *C. elegans*'**. The choice of cell division axis has consequences for proteins with regional localization, which then affects cellular organization. In early *C. elegans* embryos there are asymmetric divisions with different cell cycle times. Knowing that a G-protein $\beta$ subunit is needed to set up the axis, they used RNAi to look at the phenotypes of $\alpha$- and $\gamma$-subunit knockouts. One of the two known $\gamma$-subunits did show embryos dying of spindle defects, whilst the other gave no phenotype. None of the 20 $\alpha$-subunits were essential for spindle formation, until they found two that were substituting for each other. The G$\alpha$ and $\beta$ are normally located to the asters (centrosomes), but as yet the G protein targets are not known. In an attempt to identify the target genes, her group have initiated a genome-wide RNAi screen. For this they have generated a library of ca. 19 000 bacterial strains containing plasmids expressing an inverted duplication of a gene. Feeding the worms these bacteria targets RNAi to block translation of the message of that gene. They then score phenotypes, making a video of the first three divisions, to look for spindle defects, in terms of assembly, orientation and chromosomal segregation. So far, they have completely covered chromosome I; ca. 13% of the genes gave a phenotype and ca. 9% were embryonic lethals. One interesting observation was that genes giving a phenotype were often conserved. For example, contrast the result that only 20% of all *C. elegans* genes have an orthologue in *S. cerevisiae*, whereas ca. 50% of those genes with an RNAi phenotype had a yeast orthologue, and as many as 70% had a *Drosophila* orthologue. Of those genes giving an embryonic lethal phenotype, many were involved in transcription and translation, whereas those giving post-embryonic lethal phenotypes were commonly of unknown function. This could, however, be explained by the bias for detection of embryonic lethals in previous genetic screens.

### References

Brancia FB, Oliver SG, Gaskell SJ. 2000. Improved MALDI mass spectrometric analysis of tryptic hydrolysates of proteins following guanidination of lysine-containing peptides. *Rapid Comm Mass Spectrom (in press)*.

DeRisi JL, Iyer VR, Brown PO. 1997. Exploring the metabolic and genetic control of gene expression on a genomic scale. *Science* **278**(5338): 680–686.

McLysaght A, Enright AJ, Skrabanek L, Wolfe KH. 2000. Estimation of synteny conservation and genome compaction between pufferfish (*Fugu*) and human. *Yeast* **17**(1): 22–36.

Neubauer G, King A, Rappsilber J, *et al.* 1998. Mass spectrometry and EST-database searching allows characterization of the multi-protein spliceosome complex. *Nature Genet* **20**(1): 46–50.

The Meeting Highlights of *Comparative and Functional Genomics* aim to present a commentary on the topical issues in genomics studies presented at a conference. The Meeting Highlights are invited and each represents a personal critical analysis of the current reports and aim at providing implications for future genomics studies.

Copyright © 2000 John Wiley & Sons, Ltd. 
